# 3-Phenyl-1*H*-pyrrolo[2,1-*c*][1,4]oxazin-1-one

**DOI:** 10.1107/S1600536810017940

**Published:** 2010-05-26

**Authors:** Salman Tariq Khan, Peng Yu, Suchada Chantrapromma, Yong-En Guo, Nighat Afza

**Affiliations:** a Department of Pharmaceutical Engineering, Biotechnology College, Tianjin University of Science & Technology (TUST), Tianjin 300457, People’s Republic of China; b Crystal Materials Research Unit, Department of Chemistry, Faculty of Science, Prince of Songkla University, Hat-Yai, Songkhla 90112, Thailand; c Pharmaceutical Research Centre, PCSIR Labs Complex, Karachi 75280, Pakistan

## Abstract

The mol­ecule of the title compound, C_13_H_9_NO_2_, is slightly twisted with a dihedral angle of 4.85 (9)° between the nine-membered ring system and the phenyl ring. The nine non-H atoms of the 1*H*-pyrrolo[2,1-*c*][1,4]oxazin-1-one system are coplanar [r.m.s. deviation = 0.0122 (2) Å]. In the crystal, weak inter­molecular C—H⋯O inter­actions link mol­ecules into chains along [1
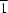
0]. The crystal studied was an inversion twin with a 0.48624 (9):0.51376 (9) domain ratio.

## Related literature

For the biological activity and applications of pyrrolo[1,2-*a*]pyrazine derivatives, see: Bélanger *et al.* (1983 ▶); Fu *et al.* (2002 ▶); Micheli *et al.* (2008 ▶). For a related structure, see: Khan *et al.* (2010 ▶). For standard bond-length data, see: Allen *et al.* (1987 ▶).
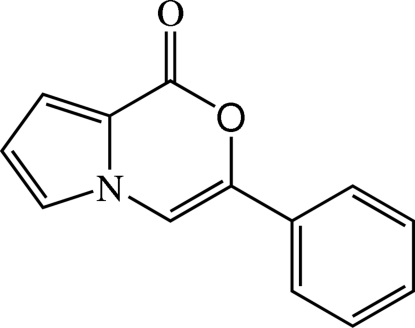


## Experimental

### 

#### Crystal data

C_13_H_9_NO_2_
*M* *_r_* = 211.21
Monoclinic, 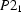

*a* = 5.870 (1) Å
*b* = 3.8345 (7) Å
*c* = 21.733 (4) Å
β = 91.059 (7)°
*V* = 489.09 (15) Å^3^
*Z* = 2
Mo *K*α radiation
μ = 0.10 mm^−1^
*T* = 113 K
0.22 × 0.18 × 0.08 mm

#### Data collection

Rigaku Saturn CCD area-detector diffractometer
Absorption correction: multi-scan (*CrystalClear*; Rigaku, 2005 ▶) *T*_min_ = 0.979, *T*_max_ = 0.992
4358 measured reflections
1222 independent reflections
1092 reflections with *I* > 2σ(*I*)
*R*_int_ = 0.032

#### Refinement

*R*[*F* ^2^ > 2σ(*F* ^2^)] = 0.031
*wR*(*F* ^2^) = 0.085
*S* = 1.10
1222 reflections
147 parameters
1 restraint
H-atom parameters constrained
Δρ_max_ = 0.20 e Å^−3^
Δρ_min_ = −0.19 e Å^−3^

### 

Data collection: *CrystalClear* (Rigaku, 2005 ▶); cell refinement: *CrystalClear*; data reduction: *CrystalClear*; program(s) used to solve structure: *SHELXTL* (Sheldrick, 2008 ▶); program(s) used to refine structure: *SHELXTL*; molecular graphics: *SHELXTL*; software used to prepare material for publication: *SHELXTL* and *PLATON* (Spek, 2009 ▶).

## Supplementary Material

Crystal structure: contains datablocks I, global. DOI: 10.1107/S1600536810017940/rz2449sup1.cif

Structure factors: contains datablocks I. DOI: 10.1107/S1600536810017940/rz2449Isup2.hkl

Additional supplementary materials:  crystallographic information; 3D view; checkCIF report

## Figures and Tables

**Table 1:** Hydrogen-bond geometry (Å, °)

*D*—H⋯*A*	*D*—H	H⋯*A*	*D*⋯*A*	*D*—H⋯*A*
C7—H7⋯O2^i^	0.95	2.27	3.109 (2)	147

Symmetry code: (i) 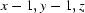.

**Fig. 1.**
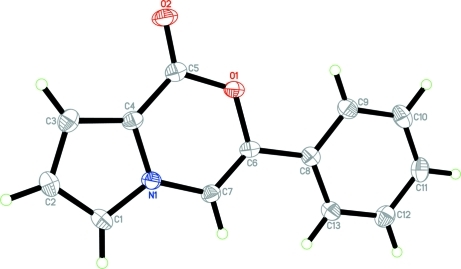
The molecular structure of the title compound, showing 50% probability displacement ellipsoids and the atom-numbering scheme.

**Fig. 2.**
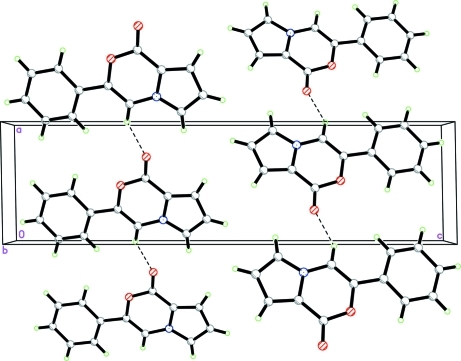
The crystal packing of the title compound viewd along the b axis. Intermoilecular C—H···O interactions are drawn as dashed lines.

## supplementary crystallographic information

### Comment

A series of pyrrolo[1,2-*a*]pyrazine compounds show potent and selective non-competitive mGluR5 antagonists properties (Micheli *et al.*, 2008). We previously reported the synthesis and crystal structure of 3-methyl-1*H*-pyrrolo[2,1-*c*][1,4]oxazin-1-one (I) (Khan *et al.*, 2010). The title compound (II), which was designed by changing the methyl substituent in (I) to phenyl, is a new key intermediate which can be used as a precursor for the syntheses of muscle relaxant agents (Bélanger *et al.*, 1983) and other biological active compounds (Fu *et al.*, 2002).

The molecule of title compound (Fig. 1) is slightly twisted, the dihedral angle between this nine membered ring system and phenyl ring being 4.85 (9)° and the O1–C6–C8–C9 torsion angle 5.0 (3)°. The nine non-hydrogen atoms of the 1*H*-pyrrolo[2,1-c][1,4]oxazin-1-one ring system are coplanar with a *r.m.s.* of 0.0122 (2) Å. The bond lengths are in normal ranges (Allen *et al.*, 1987) and comparable with the related structure (Khan *et al.*, 2010). In the crystal structure (Fig. 2), weak intermolecular C—H···O interactions (Table 1) link the molecules into chains along [110]. These chains are stacked along the *b* axis.

### Experimental

A solution of α-bromo acetophenone (2.37 g, 11.91 mmol) in acetone (25 ml) was dropwise added through a dropping funnel to a slurry of 2,2,2-trichloro-1-(l*H*-pyrrol-2-*yl*)ethanone (1.69 g, 7.95 mmol), potassium carbonate (1.98 g, 14.31 mmol) and acetone (20 ml) at room temperature in a 100 ml reaction flask. The reaction mixture was refluxed for 4 h. The solid was then removed by filtration and washed with acetone. The filtrate was concentrated under reduced pressure by rotary evaporator, the residue was partitioned between water (20 ml) and ethyl acetate (40 ml) in a separatory funnel (100 ml). The organic layer was separated and the aqueous phase was washed with ethyl acetate (30 ml x 2). The combined organic layers were washed successively with water (20 ml x 3) and brine solution and dried over anhydrous MgSO_4_. After filtration, the solvent was removed by rotary evaporator to obtain the oily residue (1.90 g) which was purified by flash column chromatography (petroleum ether:ethyl acetate, 4:1 v/v) to afford the desired compound as white solid (1.05 g, yield 62.5 %). Colourless needle-shaped single crystals of the title compound suitable for X-ray structure determination were recrystalized from ethyl acetate by slow evaporation of the solvent at room temperature after several days.

### Refinement

H atoms were placed in calculated positions with C—H = 0.95 Å, and were included in the refinement in a riding-model approximation, with *U*_iso_(H) = 1.2 *U*_eq_(C). The highest residual electron density peak and the deepest hole are located at 0.69 Å and 0.93 Å from atom C4. The crystal studied was an inversion twin, with a refined BASF ratio of 0.48624 (9)/0.51376 (9). The final refinement was carried out with Friedel pairs merged.

### Crystal data

**Table:**

C_13_H_9_NO_2_	*F*(000) = 220
*M**_r_* = 211.21	*D*_x_ = 1.434 Mg m^−^^3^
Monoclinic, *P*2_1_	Mo *K*α radiation, λ = 0.71073 Å
Hall symbol: P 2yb	Cell parameters from 1222 reflections
*a* = 5.870 (1) Å	θ = 2.8–27.0°
*b* = 3.8345 (7) Å	µ = 0.10 mm^−^^1^
*c* = 21.733 (4) Å	*T* = 113 K
β = 91.059 (7)°	Needle, colourless
*V* = 489.09 (15) Å^3^	0.22 × 0.18 × 0.08 mm
*Z* = 2	

### Data collection

**Table:**

Rigaku Saturn CCD area-detector diffractometer	1222 independent reflections
Radiation source: rotating anode	1092 reflections with *I* > 2σ(*I*)
multilayer	*R*_int_ = 0.032
Detector resolution: 14.63 pixels mm^-1^	θ_max_ = 27.0°, θ_min_ = 2.8°
ω and φ scans	*h* = −7→7
Absorption correction: multi-scan (*CrystalClear*; Rigaku, 2005)	*k* = −4→4
*T*_min_ = 0.979, *T*_max_ = 0.992	*l* = −26→27
4358 measured reflections	

### Refinement

**Table:**

Refinement on *F*^2^	Secondary atom site location: difference Fourier map
Least-squares matrix: full	Hydrogen site location: inferred from neighbouring sites
*R*[*F*^2^ > 2σ(*F*^2^)] = 0.031	H-atom parameters constrained
*wR*(*F*^2^) = 0.085	*w* = 1/[σ^2^(*F*_o_^2^) + (0.050*P*)^2^] where *P* = (*F*_o_^2^ + 2*F*_c_^2^)/3
*S* = 1.10	(Δ/σ)_max_ < 0.001
1222 reflections	Δρ_max_ = 0.20 e Å^−^^3^
147 parameters	Δρ_min_ = −0.19 e Å^−^^3^
1 restraint	Extinction correction: *SHELXTL* (Sheldrick, 2008), Fc^*^=kFc[1+0.001xFc^2^λ^3^/sin(2θ)]^-1/4^
Primary atom site location: structure-invariant direct methods	Extinction coefficient: 0.037 (9)

### Special details

**Table:**

Geometry. All esds (except the esd in the dihedral angle between two l.s. planes) are estimated using the full covariance matrix. The cell esds are taken into account individually in the estimation of esds in distances, angles and torsion angles; correlations between esds in cell parameters are only used when they are defined by crystal symmetry. An approximate (isotropic) treatment of cell esds is used for estimating esds involving l.s. planes.
Refinement. Refinement of *F*^2^ against ALL reflections. The weighted *R*-factor wR and goodness of fit *S* are based on *F*^2^, conventional *R*-factors *R* are based on *F*, with *F* set to zero for negative *F*^2^. The threshold expression of *F*^2^ > σ(*F*^2^) is used only for calculating *R*-factors(gt) etc. and is not relevant to the choice of reflections for refinement. *R*-factors based on *F*^2^ are statistically about twice as large as those based on *F*, and *R*- factors based on ALL data will be even larger.

### Fractional atomic coordinates and isotropic or equivalent isotropic displacement parameters (Å^2^)

**Table:**

	*x*	*y*	*z*	*U*_iso_*/*U*_eq_	
O1	0.4797 (2)	0.4846 (4)	0.25405 (5)	0.0201 (4)	
O2	0.7147 (2)	0.7726 (4)	0.31664 (6)	0.0273 (4)	
N1	0.1877 (2)	0.3508 (4)	0.34783 (7)	0.0181 (4)	
C1	0.0686 (3)	0.3143 (6)	0.40070 (8)	0.0219 (5)	
H1	−0.0763	0.2067	0.4044	0.026*	
C2	0.1959 (3)	0.4614 (6)	0.44782 (8)	0.0232 (5)	
H2	0.1546	0.4700	0.4899	0.028*	
C3	0.3965 (3)	0.5960 (6)	0.42329 (9)	0.0230 (5)	
H3	0.5148	0.7140	0.4453	0.028*	
C4	0.3895 (3)	0.5244 (5)	0.36106 (8)	0.0186 (4)	
C5	0.5406 (3)	0.6064 (6)	0.31182 (8)	0.0198 (4)	
C6	0.2775 (3)	0.3039 (6)	0.24323 (8)	0.0177 (4)	
C7	0.1330 (3)	0.2396 (6)	0.28844 (8)	0.0189 (4)	
H7	−0.0057	0.1192	0.2802	0.023*	
C8	0.2448 (3)	0.1991 (6)	0.17857 (8)	0.0186 (4)	
C9	0.4150 (3)	0.2573 (6)	0.13567 (8)	0.0223 (5)	
H9	0.5540	0.3651	0.1483	0.027*	
C10	0.3819 (4)	0.1584 (6)	0.07468 (9)	0.0258 (5)	
H10	0.4987	0.1992	0.0459	0.031*	
C11	0.1813 (4)	0.0015 (6)	0.05544 (8)	0.0242 (5)	
H11	0.1596	−0.0651	0.0136	0.029*	
C12	0.0115 (3)	−0.0580 (6)	0.09787 (8)	0.0248 (5)	
H12	−0.1273	−0.1651	0.0849	0.030*	
C13	0.0429 (3)	0.0377 (6)	0.15879 (8)	0.0220 (5)	
H13	−0.0738	−0.0068	0.1875	0.026*	

### Atomic displacement parameters (Å^2^)

**Table:**

	*U*^11^	*U*^22^	*U*^33^	*U*^12^	*U*^13^	*U*^23^
O1	0.0165 (7)	0.0229 (8)	0.0210 (6)	−0.0045 (6)	0.0017 (5)	0.0005 (7)
O2	0.0190 (7)	0.0308 (9)	0.0321 (8)	−0.0093 (7)	−0.0014 (5)	0.0000 (7)
N1	0.0163 (8)	0.0198 (10)	0.0183 (8)	−0.0017 (7)	0.0007 (6)	0.0014 (7)
C1	0.0206 (10)	0.0251 (12)	0.0202 (9)	0.0008 (9)	0.0047 (7)	0.0043 (9)
C2	0.0263 (10)	0.0249 (12)	0.0185 (9)	0.0048 (10)	0.0025 (7)	0.0021 (9)
C3	0.0233 (10)	0.0222 (12)	0.0234 (9)	0.0015 (9)	−0.0033 (7)	−0.0017 (9)
C4	0.0161 (9)	0.0173 (12)	0.0224 (9)	0.0000 (8)	−0.0019 (7)	0.0001 (9)
C5	0.0183 (10)	0.0170 (11)	0.0240 (9)	0.0002 (9)	−0.0017 (7)	0.0007 (9)
C6	0.0146 (9)	0.0166 (11)	0.0220 (9)	−0.0019 (8)	−0.0010 (7)	0.0017 (8)
C7	0.0172 (9)	0.0207 (11)	0.0187 (8)	−0.0025 (8)	−0.0012 (7)	0.0002 (9)
C8	0.0193 (9)	0.0168 (11)	0.0197 (9)	0.0027 (8)	0.0009 (7)	0.0020 (8)
C9	0.0202 (10)	0.0237 (12)	0.0229 (9)	0.0017 (9)	0.0011 (7)	0.0015 (10)
C10	0.0289 (11)	0.0275 (13)	0.0211 (9)	0.0049 (10)	0.0064 (8)	0.0034 (9)
C11	0.0310 (11)	0.0237 (12)	0.0178 (9)	0.0066 (9)	−0.0020 (7)	−0.0001 (9)
C12	0.0239 (10)	0.0248 (12)	0.0255 (10)	0.0016 (10)	−0.0042 (7)	−0.0020 (10)
C13	0.0199 (10)	0.0243 (13)	0.0219 (9)	0.0000 (9)	0.0030 (7)	0.0003 (10)

### Geometric parameters (Å, °)

**Table:**

O1—C5	1.380 (2)	C6—C8	1.471 (2)
O1—C6	1.391 (2)	C7—H7	0.9500
O2—C5	1.207 (2)	C8—C9	1.397 (2)
N1—C1	1.363 (2)	C8—C13	1.397 (3)
N1—C4	1.384 (2)	C9—C10	1.389 (3)
N1—C7	1.391 (2)	C9—H9	0.9500
C1—C2	1.377 (3)	C10—C11	1.380 (3)
C1—H1	0.9500	C10—H10	0.9500
C2—C3	1.400 (3)	C11—C12	1.389 (3)
C2—H2	0.9500	C11—H11	0.9500
C3—C4	1.380 (3)	C12—C13	1.383 (3)
C3—H3	0.9500	C12—H12	0.9500
C4—C5	1.438 (2)	C13—H13	0.9500
C6—C7	1.333 (2)		
			
C5—O1—C6	121.88 (14)	C6—C7—N1	119.21 (18)
C1—N1—C4	108.95 (15)	C6—C7—H7	120.4
C1—N1—C7	129.57 (17)	N1—C7—H7	120.4
C4—N1—C7	121.48 (15)	C9—C8—C13	118.58 (17)
N1—C1—C2	107.74 (17)	C9—C8—C6	120.77 (17)
N1—C1—H1	126.1	C13—C8—C6	120.65 (16)
C2—C1—H1	126.1	C10—C9—C8	120.26 (19)
C1—C2—C3	108.45 (17)	C10—C9—H9	119.9
C1—C2—H2	125.8	C8—C9—H9	119.9
C3—C2—H2	125.8	C11—C10—C9	120.78 (17)
C4—C3—C2	106.83 (18)	C11—C10—H10	119.6
C4—C3—H3	126.6	C9—C10—H10	119.6
C2—C3—H3	126.6	C10—C11—C12	119.29 (18)
C3—C4—N1	108.03 (16)	C10—C11—H11	120.4
C3—C4—C5	132.71 (19)	C12—C11—H11	120.4
N1—C4—C5	119.21 (16)	C13—C12—C11	120.47 (19)
O2—C5—O1	117.52 (16)	C13—C12—H12	119.8
O2—C5—C4	125.73 (18)	C11—C12—H12	119.8
O1—C5—C4	116.75 (16)	C12—C13—C8	120.62 (17)
C7—C6—O1	121.35 (17)	C12—C13—H13	119.7
C7—C6—C8	125.48 (18)	C8—C13—H13	119.7
O1—C6—C8	113.17 (15)		
			
C4—N1—C1—C2	−0.7 (2)	C5—O1—C6—C8	179.70 (17)
C7—N1—C1—C2	178.7 (2)	O1—C6—C7—N1	−1.1 (3)
N1—C1—C2—C3	0.9 (2)	C8—C6—C7—N1	179.16 (18)
C1—C2—C3—C4	−0.7 (2)	C1—N1—C7—C6	−179.8 (2)
C2—C3—C4—N1	0.3 (2)	C4—N1—C7—C6	−0.5 (3)
C2—C3—C4—C5	177.6 (2)	C7—C6—C8—C9	−175.2 (2)
C1—N1—C4—C3	0.2 (2)	O1—C6—C8—C9	5.0 (3)
C7—N1—C4—C3	−179.20 (19)	C7—C6—C8—C13	4.5 (3)
C1—N1—C4—C5	−177.49 (18)	O1—C6—C8—C13	−175.29 (19)
C7—N1—C4—C5	3.1 (3)	C13—C8—C9—C10	0.5 (3)
C6—O1—C5—O2	−177.16 (18)	C6—C8—C9—C10	−179.8 (2)
C6—O1—C5—C4	2.6 (3)	C8—C9—C10—C11	0.0 (3)
C3—C4—C5—O2	−1.3 (4)	C9—C10—C11—C12	−0.1 (4)
N1—C4—C5—O2	175.7 (2)	C10—C11—C12—C13	−0.2 (4)
C3—C4—C5—O1	178.9 (2)	C11—C12—C13—C8	0.7 (4)
N1—C4—C5—O1	−4.0 (3)	C9—C8—C13—C12	−0.8 (3)
C5—O1—C6—C7	−0.1 (3)	C6—C8—C13—C12	179.4 (2)

### Hydrogen-bond geometry (Å, °)

**Table:**

*D*—H···*A*	*D*—H	H···*A*	*D*···*A*	*D*—H···*A*
C7—H7···O2^i^	0.95	2.27	3.109 (2)	147

Symmetry codes: (i) *x*−1, *y*−1, *z*.

## References

[bb1] Allen, F. H., Kennard, O., Watson, D. G., Brammer, L., Orpen, A. G. & Taylor, R. (1987). *J. Chem. Soc. Perkin Trans. 2*, pp. S1–19.

[bb2] Bélanger, P. C., Atkinson, J. G., Rooney, C. S., Britcher, S. F. & Remy, D. C. (1983). *J. Org. Chem* **48**, 3234–3241.

[bb3] Fu, D.-C., Yu, H. & Zhang, S.-F. (2002). *Chin. Chem. Lett* **13**, 1051–1054.

[bb4] Khan, S. T., Yu, P., Hua, E., Ali, S. N. & Nisa, M. (2010). *Acta Cryst.* E**66**, o711. 10.1107/S1600536810006951 PMC2983595 21580449

[bb5] Micheli, F., Bertani, B., Bozzoli, A., Crippa, L., Cavanni, P., Di Fabio, R., Donati, D., Marzorati, P., Merlo, G., Paio, A., Perugini, L. & Zarantonello, P. (2008). *Bioorg. Med. Chem. Lett* **18**, 1804–1809. 10.1016/j.bmcl.2008.02.024 18304814

[bb6] Rigaku (2005). *CrystalClear* Rigaku Corporation, Tokyo, Japan.

[bb7] Sheldrick, G. M. (2008). *Acta Cryst.* A**64**, 112–122. 10.1107/S0108767307043930 18156677

[bb8] Spek, A. L. (2009). *Acta Cryst.* D**65**, 148–155. 10.1107/S090744490804362X PMC2631630 19171970

